# Annual Report of the Supervising Surgeon of the Marine Hospital Service of the United States

**Published:** 1874-06

**Authors:** 


Annual Report of the Supervising Surgeon of the Marine Hospital Service of the United States. For the fiscal year 1873. *Complimentary*, by John M. Woodworth, M.D.

This is a valuable volume. Besides the Report proper, which is devoted, among other things, to "a summary statement of observations U. S. Marine Hospital service; statement of hospital relief; table of diseases treated in Hospital, etc.," it contains an appendix with the following valuable contributions: Hospitals and Hospital Construction, by J. M. Woodworth, M.D.; Natural History of Yellow Fever in the United States, by J. M. Toner, M.D.; Yellow Fever Epidemic; Reports from Medical Officers; Report of a Case of Double Diaphragmatic Rupture of Hernia, by Thomas T. Minor, M.D.; Urethral Strictures, by C. N. Ellinwood, M.D., etc. It is a handsome volume, and contains several good illustrations and maps.

